# Improvements in the quality of the parent-child relationship following treatment with an integrated family approach

**DOI:** 10.3389/fpsyt.2024.1377100

**Published:** 2024-06-28

**Authors:** Hanna Stolper, Lieke Imandt, Karin van Doesum, Majone Steketee

**Affiliations:** ^1^ Department of Psychology Education and Child Studies, Erasmus University Rotterdam (EUR), Rotterdam, Netherlands; ^2^ Jeugd ggz, Dimence Groep, Zwolle, Netherlands; ^3^ Radboud University Nijmegen, Department of Clinical Psychology, Nijmegen, Netherlands; ^4^ Impluz, Dimence Groep, Deventer, Netherlands; ^5^ Verwey-Jonker Instituut, Utrecht, Netherlands

**Keywords:** integrated family approach, family focused practice, adult and child mental health services, parental mental disorder, infants and early childhood, intergenerational transmission of mental disorders, emotional availability scales, parental reflective functioning

## Abstract

**Objective:**

This study investigated changes in the emotional availability of the parent and the child in the dyadic relationship, parental reflective functioning, and parental perception of the relationship with their child following treatment with an integrated family approach in adult and child mental health care services. The aim of the study was to investigate if an integrated family approach in treatment contributes to good practice in mental health care.

**Background:**

Children of parents with a mental disorder are at increased risk for developing mental health problems themselves during lifetime. Infants are extremely vulnerable for environmental influences. Parents with mental disorders are at risk for mis-attuned behavior and non-optimal emotional availability. This increases the risk of adverse cascading effects on the parent-child relationship and child development. A secure parent-child relationship is an important protective factor against the intergenerational transmission of mental disorders. Although treatment of the parental mental disorder is important, it does not automatically change undesirable patterns in the parent-child relationship. Therefore, an integrated family approach to mental health treatment is recommended.

**Methods:**

This study involved a mixed methods design using questionnaires, an observation instrument and semi-structured interviews. The variables examined were the quality of the parent-child interaction, the parental perspective on their relationship with the child, their problems with child upbringing, and on their parental reflective functioning. The clinical sample consisted of 50 patients with a variety of mental disorders and their young children.

**Results:**

After finishing the integrated treatment the quality of the parent-child interaction had improved significantly. Likewise, we found a significant improvement in parental perception regarding the relationship with the child and the parental role. The majority of the parents interviewed showed that they were better able to mentalize about themselves, their child and their relationship with the child, but the data from the questionnaire showed mixed results regarding parents’ reflective functioning.

**Conclusion:**

Treatment with an integrated family approach to mental health care in which the parental role of the patient, the young child, and the parent-child relationship are integrated in treatment, can be a valuable addition to the current practice of mental health care in which patients are commonly perceived as individuals.

## Introduction

Epidemiological research has found convincing evidence that offspring of parents suffering from mental disorders are at increased risk for developing mental disorders themselves (e.g. 1, 2). The parent-child relationship is in general the first and most influential relationship in a child’s early life. Parents with mental disorders may be impeded in caring for their young child by being preoccupied with their own concerns and with managing the symptoms associated with their mental dysregulation. This constitutes a risk to developing a healthy parent-child relationship, with consequences for the parent and the child. For most parents, parenthood has great significance when it comes to the personal fulfillment of life’s meaning. For the child, a healthy parent-child relationship provides a secure foundation for ongoing development and a buffer against the development of mental disorders. The importance of the stage of infancy for later development and the challenging role of parenting, especially for parents suffering from mental disorders, warrants an integrated family approach in mental health care. By this, we mean an approach in which the development of the parent-child relationship is included in the treatment of the parents’ mental disorder (3). In this study we will evaluate whether the parent-child interaction was improved among parents and young children when an integrated family approach was used in their treatment.

Over the last 12 years, a Dutch mental health care service named Dimence Groep has built up expertise with regards to an integrated family approach in treatment (4–6). This means multidisciplinary treatment is provided by a network of professionals from adult mental health services (AMHS) and child and adolescent mental health services (CAMHS), all embracing an integrated family approach in their treatments. The involved professionals meet regularly for multidisciplinary consultations, share their perspectives and experiences, and tailor the treatment components to the needs and capabilities of the family. The aim of this integrated treatment is to improve the relationship between parents and their young child in order to protect them from the consequences of intergenerational transmission of mental disorders and adverse outcomes.

Although treatment of the parental mental disorder is important, it will not automatically change undesirable patterns in the parent-infant relationship (7–9). To foster the resilience of young children whose parents have been diagnosed with a mental disorder the focus needs to be on the parent-child system (10, 11). There is some preliminary support in clinical samples that suggests an association between higher levels of insecure attachment in infants and parental behaviors related to mental disorders (12, 13). A secure attachment relationship between child and parent is perceived as an important protective factor against the development of mental disorders (10, 14, 15). Doty et al. (16), argued that a positive development or change in the parent-child relationship will have positive spillover effects over time on other domains impacting the parent (parental efficacy, positive emotions) as well as the child (cognitive, emotional and social functioning).

The emotional availability of parents is an important predictor of the child’s secure attachment. However, this may not be evident for parents with a mental disorder (12, 17). The concept of emotional availability (EA), grounded in attachment theory, was initially focused on the parent’s sensitivity to the child’s emotional signals and later supplemented by the child’s emotional availability towards the parent (18). The latter means the emotional signals made by the child to the parent (e.g. smiling, crying, seeking or terminating eye contact). The child’s EA is of importance for the parent to be able to understand what the child wishes to communicate to the parent about his or her needs. With this expansion of the theory, EA developed into a dyadic concept and was therefore consistent with Sameroff’s (19) transactional model in which the parent-child relationship is conceived as a reciprocally shaped system. Regarding the concept of EA, the child’s emotional availability to the parent should also be addressed.

In various research, it is demonstrated that parents’ capacity to mentalize is also an important predictor of the child’s secure attachment (20, 21), and resilience to adversity (20). Parental mentalization or parental reflective functioning (PRF) refers to the parents' capacity to understand their own as well as their child’s behavior as related to internal mental states such as thoughts, feelings, and wishes (22–24). Some empirical evidence points to a two- or three dimensional structure of PRF:

1. Self-focused reflective functioning - the parental capacity to mentalize about their own emotions, feelings, and behaviors,
2. Child-focused reflective functioning - the parental capacity to mentalize about the emotions, feelings (mental states), and behaviors of the child (25–27), and
3. Relation-focused reflective functioning - the (parental) capacity to mentalize about how dynamics of mental states (both parent and child) affect the interactions and behavior in relationships (with the child) (27).

Parental mentalization allows parents to be sensitive, meaning that they can accurately perceive and interpret the infant’s signals and communications and respond appropriately. Consequently, the parent is able to see the child as a separate individual with his or her own emotions, feelings and wishes (23, 28). Regulating and comforting the child in a sensitive and an appropriate way plays a vital role in the development of attachment and the child’s self-regulation and capacity to mentalize (22). A meta-analysis (21) of parental mentalization and sensitivity as predictors of infant attachment found a direct effect of parental mentalization on infant attachment, over and above parental sensitivity, as well as an indirect effect on parental sensitivity mediating the relationship between parental mentalization and the infant’s attachment security.

Parental reflective functioning may perform as a protective factor for risk factors such as parental mental disorder (e.g. trauma) and disruptive parental behavior. These factors are associated with a child’s outcomes, such as insecure attachment (20). Therefore, enhancing secure attachment between parent and child, and thus focus on parental mentalization and parental sensitivity to the child’s cues, are considered to be critical targets for intervention (21, 29). Moreover, Nijssens et al. (30) conclude that these intervention targets could have a preventive function with regard to the social-emotional development of young children. In practice, however, these factors are little considered in the treatment of adults with mental health problems who have young children.

## Current study

In this mixed method study, we will evaluate the outcomes of an integrated family approach in treatment on the quality of the parent-child interactions. With this in mind, we will focus on two features of the parent’s EA - sensitivity and non-intrusiveness to the child - and on the EA of the child to the parent, including their responsiveness and involvement. Furthermore, we will investigate the parental perspective on their relationship with the child, their problems with child upbringing, and on their parental reflective functioning. Based on the literature (8, 9, 31), we hypothesized that the integrated family approach would lead to an improvement in the quality of the EA of the parent and the child in the parent-child interaction, and an improvement in parental mentalization. The aim of the study is to test the expected improvements of an integrated family approach on parent-child interaction and parental mentalization in order to contribute to good practice in mental health.

The research questions were:

1. Is there improvement in the quality of the parent-child interaction for patients who receive treatment with an integrated family approach? We hypothesized the parent-child interaction to improve from pre to post on average.
2. Is there a change in the parental perspective on the relationship with the child and on problems with the child’s upbringing? We hypothesized a positive change in the parents’ perception of the relationship with their child and a reduction of problems in child’s upbringing.
3. Is there improvement in parental reflective functioning for patients who have received treatment with an integrated family approach? We hypothesized parental mentalization to improve over the period of treatment.

By finding answers to these research issues we aim to contribute towards improving the development of the early parent-child relationship between patients treated in mental health care services and their children.

## Materials and methods

### Design

The current study consisted of a mixed methods design with quantitative and qualitative data. Quantitative data was collected from an observation instrument and questionnaires in a pre and post-measurement design without a control group. The qualitative data was collected from semi-structured interviews with parents in the post-measurement.

### Sample

Participants were recruited at the department of adult mental health services (AMHS) and child and adolescent mental health services (CAMHS) within the Dimence Groep, a mental health care foundation in The Netherlands. The number of potential participants was 110, but 43 of them refused to participate. The most commonly mentioned reason given in this regard by patients was the stress they were experiencing. A few patients expressed no confidence in the privacy statement provided for the research or expressed fear of the involvement of child protection services. All adult patients and their young children up to six years were diagnosed by a psychiatrist, or a psychologist, respectively according to the DSM-5 and the Diagnostic Classification of Mental Health and Developmental Disorders of Infancy and Early Childhood (DC:0–5™).

To avoid the false impression of a homogenous group and stay close to the reality of daily clinical practice, where patients demonstrate a high variety of mental disorders, differences in comorbidity, and a considerable heterogeneity phenomenology of mental disorders, we did not focus on a specific DSM-5 classification. Thus, there were no exclusion criteria for the assigned DSM-5 classifications either for the parents or the infants. The study included patients and their young children who were referred to an integrated family approach for treatment due to concerns about the parents’ mental health and its impact on parenting, as well as concerns about the emotional development and mental health of the child. The group of adult patients consisted of 80% women and 20% men, with an average age between 25 and 35, and all of them had chronic and complex mental disorders. Among the adults, 64% had one or more comorbid diagnose(s) and 80% had been treated at least once in a mental health care service before. Twenty percent of the adult patients had a low level of education. The duration of treatment was predominantly longer than 12 months. The group of children consisted of approximately 50% boys, and 50% girls with a mean age of 22 months (SD = 20). Fifty-two percent of the children were under the age of 12 months at the time of referral. All of the children had at least one classification on the DC:0–5 comparable to DSM-5. In 78% of cases, parent-child relational problem was the primary classification. The characteristics of the parents and the children are shown in **Tables 1**, **2**.

**Table 1:** Characteristics of adult patients (N = 50).

		N	%
Gender	Man	9	18
	Woman	41	82
Age	<30	20	40
	30–35	17	34
	>35	13	26
Highest educational level attained	Low (basic or pre-vocational secondary education)	10	20
	Middle (secondary vocational education)	26	52
	High (bachelor or master degree)	14	28
Classification DSM-5 (only first classification)	Personality Disorder	15	30
	Bipolar Disorder	1	2
	Depressive Disorder	7	14
	Anxiety Disorder	5	10
	Autism Spectrum Disorder	6	12
	Trauma and Stressor-Related Disorder	11	22
	Others	5	10
	Comorbidity	32	64
Number of previous treatments in mental health care (adult patients)	First treatment	10	20
	Second treatment	14	28
	More than two treatments before	26	52
Duration of treatment with an integrated family approach (months)	0–6	5	10
	6–12	11	22
	12–18	12	24
	18–24	8	16
	> 24	14	28

**Table 2:** Characteristics of children (N = 50).

		N	%
Gender	Boy	24	48
	Girl	26	52
Age on time of referral (months)	0–12	26	52
	12–24	7	14
	24–36	2	4
	36–48	8	16
	48–60	4	8
	60–66	3	6
Family structure	Both biological parents	34	68
	One biological parent	10	20
	Post-divorce co-parenting	4	8
	Fosterparents	2	4
Classification DSM-5* (only first classification)	Autism Spectrum Disorder	2	4
	Unspecified Neurodevelopmental Disorder	4	8
	Post-Traumatic Stress Disorder	4	8
	Parent-Child Relational Problem	39	78
	Other	1	2
	Comorbidity	12	44

*Comparable with the classifications of the DC:0–5™.

Due to the heterogeneity of the sample, a standard treatment protocol was not utilized. However, the sample shared a commonality in the presence of a mental disorder in the parent and problems in the parent-child relationship. The treatment interventions of AMHS and CAMHS were combined and focused on addressing these two aspects. At AMHS, the parent’s mental disorder was treated with psychotherapy (individual or group), trauma therapy, psychomotor therapy, emotion regulation training, pharmacotherapy, or a combination of these interventions. At CAMHS, the treatment focus was primarily on improving the quality of the parent-child relationship through interventions such as parent-child psychotherapy, parent counseling, home treatment, or a parent-child psychotherapy group. If the child had a specific psychological disorder, such as trauma or autism spectrum disorder, specific treatment was offered. This included trauma therapy in collaboration with the parent, psychoeducation for the parent about the specific disorder, pharmacotherapy, or a combination of these interventions. If there were any issues within the family, such as problems in the couple’s relationship or specific challenges with the child’s upbringing, the treatment plan was expanded to include interventions aimed at addressing those specific issues. For a detailed description of all the possible interventions of AMHS and CAMHS within an integrated family approach in treatment (6).

### Procedure

Participants were recruited between January 2018 and May 2023. Ethics approval was granted by the Medical Ethics Review Board at the University Medical Centre of Utrecht in the Netherlands (18–186/C). During the recruitment period, all parents of children up to six years old who were in treatment with an integrated family approach were informed about the study by their therapists at either AMHS or CAMHS. Formal consent for participation was obtained by signing a consent form. The signed statement of consent was stored in the case file. All parents could withdraw from the study at any time without affecting the continuation of their treatment.

The pre-measurement took place when the integrated family approach in treatment started and post-measurement after ending this treatment. In both measurements the registered parent and the child were filmed for twenty minutes, the recommended minimum length (18), during a contact moment in a child friendly room at the office or at home. The instruction here was, “Do as you are used to doing with your child”. No specific instruction was given, and the researcher did not participate in the interaction. In addition, the enrolled parent was asked to complete two digital questionnaires: one regarding parental reflective functioning and the other regarding their perception of the parent-child relationship. After the integrated treatment was completed, individual interviews were conducted with several parents about their experiences with an integrated family approach in their treatment. To answer the research questions regarding the perception of parents about the quality of the relationship with their child, and their own parental mentalization after the treatment, we made use of data from the two questionnaires and semi-structured interviews of a previously conducted multiple case study (5), which was linked to this study. At the post-measurement, the parents received a small gift for their child.

### Measurements

The quality of the parent-child interaction was assessed using four subscales related to the parent-child interaction of the *EAS (Emotional Availability Scales), 4th edition* (32). The EAS is an observation instrument that does not quantify distinct behaviors but analyzes the interactional *style* of the dyad. It is an emotion-focused measure that refers to the overall affective quality of the relationship. The construct of emotional availability (EA) is multidimensional, as it comprises different dimensions of caregiving (33). The EAS-IV is widely used and has predictive and concurrent validity with several attachment measures. Construct validity has been established in longitudinal studies and multi-cultural populations (34). Short-term test-retest reliability is moderately strong for the parent dimensions sensitivity and non-intrusiveness (35). The tool uses video data of ≥20 min to assess EA cross parent and child scales. In this study two parent scales, Sensitivity and Non-intrusiveness, and two child scales, Responsiveness and Involvement, were used. For each scale, a direct global score is generated on a Likert scale (1 = *nonoptimal* to 7 = *optimal)* and a total score is generated using seven subscales (range 7–29) (34, IMH Journal, January 2022). Total sum scores above 20 on the subscales imply a sufficiently positive interaction between parent and child and a sufficient engagement with each other (32). Couples of two EAS-IV professionals, trained by the author of the scales, blind-coded the video data without preliminary information about the family and if it was a pre or post-measurement. Intra-class correlations (ICC, two-way random with absolute agreement, mean values and a 95% confidence interval) of .65 to .77 for the mean of the total subscale scores of both professionals indicated substantial inter-rater reliability.

The parent’s perception of the parent-child relationship was measured using the subscales Parent-child Relationship Problems (6 items) and Parenting Problems (7 items) of the digital *Parenting Stress Questionnaire* (*PSQ*; 36). This questionnaire consists of 34 questions measuring different aspects of parenting stress. In this study, the raw sum scores of the above subscales were used after mirroring the scores as described in the manual. The Parent-Child Relationship Problems subscale refers to the extent to which the parent or caregiver experiences problems in the relationship with the child. The Parenting Problems subscale indicates whether the parent feels that he or she has (in)sufficient skills to raise the child. Each item is rated on a four-point Likert scale: from 1 meaning “does not apply” to 4 “applies entirely”. The reliability of the PSQ is described as good (37). Cronbach’s alpha for the total score ranges from .89 to .91, and for the scale scores range from .74 to .87. In this study, Cronbach’s alpha was .94 for T1 and .87 for T2 for the Parent-Child Relationship Problems subscale at 45 measurements. For the Parenting Problems subscale, Cronbach’s alpha was .90 for T1 and .83 for T2 at 45 measurements.

All three subscales of the *PRFQ* (*Parental Reflective Functioning Questionnaire*; 24), a brief, multidimensional self-report measure, were used to assess the parent’s perception of parental reflective functioning (PRF), defined as the caregiver’s capacity to reflect upon his/her own internal mental experiences as well as those of the child. Higher PRF is associated with adequate caregiving and the child’s attachment security and mental health (38, 39). The PRFQ is an 18-item questionnaire that includes items related to parental interest and curiosity about their child’s mental states and how these mental states may have an impact on behavior.

Luyten et al. (24) developed three subscales to capture key features of PRF. First, there is the Pre-Mentalizing Modes (PM) subscale, in which the items capture a nonmentalizing stance, malevolent attributions, and an inability to enter the subjective world of the child (e.g., “My child cries around strangers to embarrass me”). The second is the Certainty about Mental States (CMS) subscale: scores on this scale may range from hypermentalizing, meaning a tendency of parents to be overly certain about the mental states of their child (i.e., to not recognize the opacity of mental states), to hypomentalizing, that is, an almost complete lack of certainty about the child’s mental states (e.g., “I always know what my child wants”). Thirdly, there is the Interest and Curiosity (IC) subscale, which relates to parental interest in and curiosity about mental states, a key factor in PRF (e.g., “I am often curious to find out how my child feels”). Whereas low levels on the IC subscale might reflect an absence of interest in one’s child's mental states, very high scores might reflect intrusive hypermentalizing. Each subscale consists of 6 items and each item is rated on a 7-point Likert scale, from “1” to express “strongly disagree” to “7” to indicate “strongly agree”. The PRFQ has no well-established clinical cut-offs for its subscales. For the PM subscale, higher scores indicate lower levels of parental reflective functioning. For the CMS and IC subscales (slightly) above middle scores (i.e., M ~ 3.8 – 4 for CMS and M ~ 5.5 - 6.0 for IC in community mothers and fathers) may be more optimal, whereas either low or very high levels may be more dysfunctional (40–42). The PRFQ exhibits good construct validity, internal consistency for all subscales and reliability (24, 39). Cronbach’s alpha for this sample ranged from .58 (T1) to .63 (T2) for PM, from .65 (T1) to .73 (T2) for IC and was .72 for CMS (both T1 and T2).

For this study 18 semi-structured interviews of parents were used from the previous study (5). We adopted a qualitative design with thematic analysis, which is suitable for identifying common and overarching themes. We used Atlas-ti 8 software for coding. An extensive description of the methods can be found in the forementioned paper (5). The following two categories of the codebook constructed for the analysis of the interviews were selected: 1. parents’ comments about experienced improvement of the relationship with their child after treatment; 2. parents’ comments in which they demonstrated their ability to mentalize about themselves, their child and the relationship between them after treatment.

**Figure 1:**
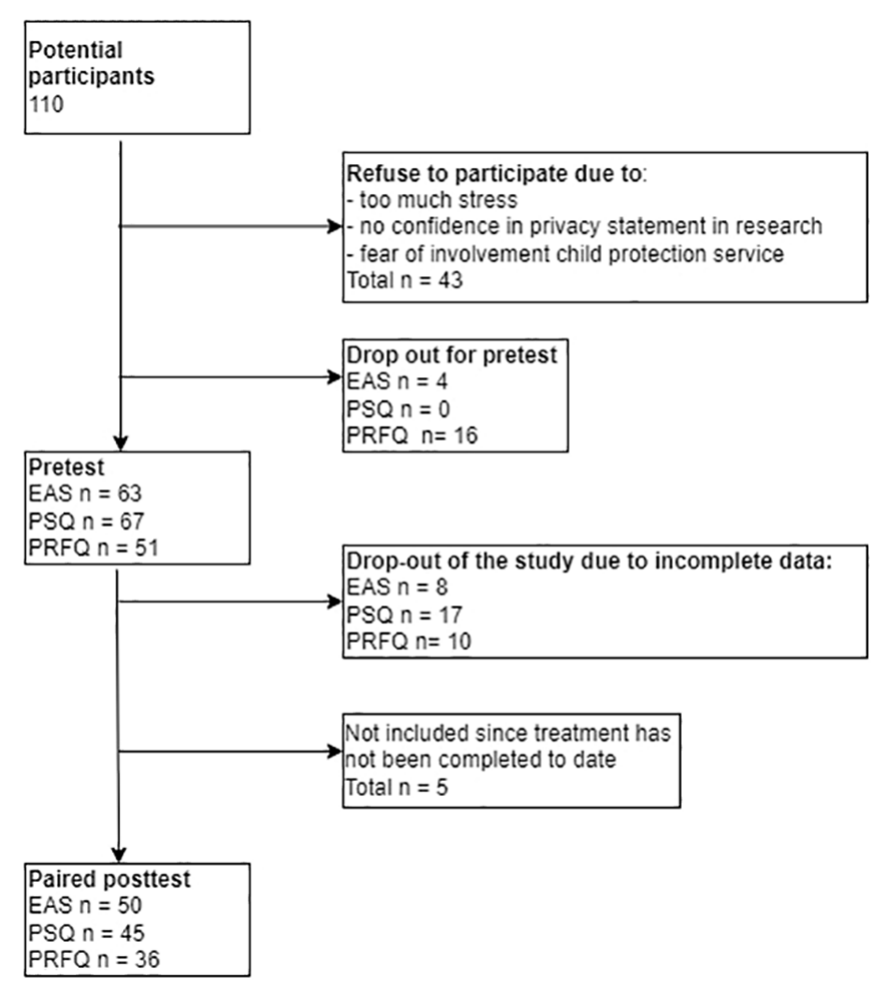
Overview of the process of data collection and dropouts of the measurements on EAS, PSQ, and PRFQ.

**Figure 1** shows an overview of the process of data collection and dropouts of the measurements on EAS, PSQ, and PRFQ.

### Data analysis

#### Power calculations and sample size

The total sample size was calculated to detect a medium sized effect in the outcome measures of parent-child interaction (EAS-IV), the parents perception of the parent-child relationship (PSQ) and of parental reflective functioning (PRFQ) at T1 and T2, utilizing G*Power software (43). A sample of 27 (number of pairs) was required to achieve a power of 80% and a level of significance of 5% (one sided), for detecting an effect size of .5 (Cohen’s *d)* between pairs.

#### Statistical analysis

For all data, the assumptions for normality and skewness were checked. To detect changes in the quality of parent-child interaction, the parents perception of the parent-child relationship, and of their certainty of mental states, the paired sample *t*-test was used. The Wilcoxon signed rank test was used to detect changes in the parents perception of prementalizing (PM) and interest and curiosity (IC). Pre and post-test-scores on CMS and IC were compared with the optimum score. Using the standard error of difference (*S_diff_*), the Reliable Change Index (RCI) was calculated to examine whether the differences found per participant were statistically significant (44, 45). An RCI equal to or greater than 1.96 is considered statistically significant. Statistical analyses were conducted in SPSS version 27.

#### Qualitative data analysis of the interview questions

The current study is linked to a multiple case study evaluating the use of an integrated family approach in treatment in which parents were interviewed about their experiences with this approach in treatment (5). An extensive description of materials and methods as well as the offered treatment are described in this previous publication.

## Results

All data from the EAS and PSQ were normally distributed and showed statistically and clinically significant results. The PRFQ showed mixed results. Only the CMS-subscale was normally distributed. For a small group of the participants the subscales scores of the three scales of the PRFQ moved to the optimal score. Results from the quantitative and qualitative analysis are reported below and the quantitative results are presented in **Table 3**.

**Table 3:** Results of the paired samples t-test for quality of the parent-child interaction, parenting problems and parental mentalization.

Variable	Premeasurement score (T1) M SD	Postmeasurement score (T2) M SD	Difference M SD	95% conf. interval of the difference from to		p-value	S_diff_	% improved based on RCI	Effect Size
Sensitivity (*N* = 50)	18.85 3.62	21.02 2.96	2.17 3.85	1.08	3.27	.000**	.66	62	.56
Non-intrusiveness (*N* = 50)	19.47 3.98	21.02 2.83	1.55 4.17	.36	2.73	.012*	.67	56	.37
Responsiveness (*N* = 50)	18.91 3.41	21.37 3.26	2.47 4.49	1.19	3.74	.000**	.67	60	.54
Involvement (*N* = 50)	18.23 3.39	20.85 3.47	2.62 4.27	1.41	3.83	.000**	.68	68	.61
Parent-Child Relationship Problems (*N* = 45)	13.69 4.73	10.96 3.74	2.73 4.55	1.36	4.10	.000**	.87	53	.59
Parenting Problems (*N* = 45)	17.11 4.48	14.76 3.68	2.36 4.97	.86	3.85	.003*	.86	56	.54
Prementalizing (*N* = 36)	2.42 .85	2.17 .86	-.25 1.14	-.64	.14	.19^a^	.20	38	.22^b^
Certainty of Mental States (*N* = 36)	3.70 .98	4.13 .89	.42 .88	.13	.72	.007**	.22	33	.48
Interest and Curiosity (*N* = 36)	5.48 .87	5.60 .80	.13 .91	-.18	.43	.42^c^	.19	22	.14^d^

*p<.05; **p<.01.

a results of the Wilcoxon signed-rank test: Z = -1.59, p = .11.

b r (Z/√N) = .27.

c results of the Wilcoxon signed-rank test: Z = -.49, p = .63.

d r (Z/√N) = .08.

### Quality of the parent-child interaction measured with the EAS

After a period of treatment with the integrated family approach (*µ* = 12–24 months), both the parent and child scales showed significant improvement (Parental Sensitivity: *t*(49)= 3.99, *p* =.000, Hedges’*g* = .56; Parental Non-intrusiveness: *t* (49)= 2.62, *p* = .012, Hedges’*g* = .37; Child Responsiveness: *t*(49)= 3.88, *p* = .000, Hedges’*g* = .54; Child Involvement: *t*(49)=4.34, *p* = .000, Hedges’*g* = .61).

The RCI also showed a clinically relevant change in the quality of parent-child interaction. At the individual level, 62 percent of the participants scored better on the Sensitivity subscale of the EAS at the post-measurement (RCI ≥ 1.96). For the Non-intrusiveness, Responsiveness and Involvement subscales this was 56, 60 and 68 percent respectively. For two-thirds of the participants a total score of 20 points or above was given on the four subscales at the post-measurement, which implies a sufficiently positive interaction between parent and child. At the pre-measurement this was the case for 40 (Sensitivity), 38 (Non-intrusiveness), 32 (Responsiveness) and 22 (Involvement) percent of the participants.

### Parent’s perception of the parent-child relationship and problems with upbringing measured with the PSQ

After treatment with the integrated family approach, parents experienced fewer problems in the parent-child relationship and with raising their child (Parent-child relationship problems: *t*(44)= 4.03, *p* = .000, Hedges’*g* = .59; Parenting problems: *t*(44)= 3.18, *p* = .003, Hedges’*g* = .54). For more than half of the participants the RCI also showed a clinically relevant change in the problems parents experienced in their relationship with their children (RCI ≥ 1.96). At the post-measurement, 53 percent of the parents reported significantly fewer problems in the parent-child relationship, and 51 percent of the participants scored in the range for no to mild problems, where this was 22 percent at the pre-measurement. Fifty-six percent of the parents reported significantly fewer problems with parenting at the post-measurement. However, only 11 percent scored in the range for no to mild problems where this was 6 percent at the pre-measurement.

### Parent’s perception of parental reflective functioning measured with the PRFQ

The paired samples *t*-test showed that parents rated a significant change in their certainty about their child’s mental states over the treatment period (*t*(35)= 2.88, *p* = .007, Hedges’ *g* = .48). The Wilcoxon signed-rank test showed that treatment with the integrated family approach did not elicit a statistically significant change in the parents’ perception of pre-mentalizing (*Z* = -1.59, *p* = .11, *r* = .27) and interest and curiosity (*Z* = -.49, *p* = .63, *r* = .08). At the individual level 38 percent of participants scored significantly better on the PM subscale at the post-measurement (RCI ≥ 1.96). Disregarding the optimal score, there was a clinically relevant change in 67 percent of cases for CMS and IC. For CMS, the score moved at least half a point toward the optimal score for 33 percent of the participants. For IC, this was the case for 22 percent of the participants.

### Parents’ perception about the improvement in the quality of the parent-child relationship and their parental mentalization measured by interviews

Almost all of the 18 parents reported positive changes regarding the way they experienced the relationship with their child and their own parental mentalization after completing treatment with the integrated family approach. Three parents reported no improvements in the relationship with their child and reported no changes in their ability to empathize with their child. The first mother mentioned that the relationship with her son had not improved, but the relationship with her partner had improved through couples therapy, and she felt satisfied with his support. She also showed an awareness of the reciprocity of the relationship with her son. The second mother mentioned that she always had experienced the relationship with her son as very good, and the third mother felt that the treatment neither improved nor worsened the parent-child relationship.

In the next section, we will report on what parents told us about what they learned and what improvements they observed in the relationship with their child. Staying close to the content and analysis of the interviews, we will illustrate the emerged themes with quotations. After each quotation, we will refer to the theoretical dimensions of EA and PRF as explained earlier. See **Table 4** for an overview of the themes in the interviews with the parents. 

**Table 4:** Themes in the interviews with parents about changes in the interactions in the parent-child relationship and parental mentalization after completing the treatment.

Improvements about the behavior of the child in the parent-child interaction
Changes in parent’s behavior towards the child
Parent’s awareness about the impact of their own behavior on the child
Parent’s ability to understand and empathize with the child
Parent’s capability to regulate their own emotions, feelings, and thoughts
Parent’s capability to observe their child
The power of parental self-confidence

In terms of reported *improvements about the behavior of the child in the parent-child interaction*, parents expressed that the child showed more comfort seeking behavior toward the parent, such as proximity and cuddling (involvement).

A mother reported the following improvements she observed regarding the behavior of her son:

*[Now] when he is feeling sad, he will come and sit on my lap all cuddling and crying*. [child involvement].

Another mother said:

*She [the child] relies [on me] much more than she did as a young baby. So, that is really nice to observe.* [child involvement].

Regarding their *own parental behavior towards the child*, parents reported an increased ability to pay attention to the child’s needs (sensitivity), for example by comforting the child, by being available to the child, or by allowing the child’s play to unfold autonomously (non-intrusiveness).

A mother said:

*When she [the child] expressed that she wanted to play with me, I don’t take the lead, because I did that before. When I took the lead before, yes, of course she got a little angry and irritated. But I didn’t realize that I was directing.* [non-intrusiveness, self-, child-, and relation-focused mentalizing].

Another mother said that she learned to adjust her behavior towards the needs of her son:

*Now I put clear boundaries because we found out that he just needs that too.* [child-focused mentalizing].

Parents also reported *more awareness about the impact of their parental behavior on the child* (relation-focused mentalization). For instance, they mentioned that their emotional availability and sensitivity to the child had a positive impact on the child’s responsiveness.

A father said the following:

*As I started doing more with him, I noticed that there was more interaction. That as a baby he began to look for me more.* [parental emotional availability, child’s responsiveness].

Another father explained:

*I can be much more on his level when playing or something. Instead of sort of killing it by saying ‘yeah you know…’. A simple example: he had imagined that the ambulance was coming to put out the fire, and instead of letting his imagination flow, which gave him a lot of enjoyment, I would say, ‘Yeah, why, an ambulance can’t put out a fire’. And then you could see the whole enjoyment and fun going away and now I can think ‘ah okay, fine boy, we’ll go out with the ambulance. Very good’*. [self-, child-, and relational-focused mentalizing].

Many parents mentioned that throughout the treatment their ability *to understand and empathize with the child* (child-focused mentalization) increased, which made it easier to be sensitive to the child’s needs. They were better able to avoid the projection of their own problems and tension on the child and felt less rejected by their child (self-focused mentalization). They were more able to see the child as a person with its own needs, thoughts and feelings (child-focused mentalization).

One mother explained:

*If she wants to play with me, then we play, and if she wants to play alone then she simply plays alone. At first, I felt really bad about that. I just interpreted that very personally: that she doesn’t want to play with me. But now I feel like ‘well if she doesn’t want to play with me, I should just let her*’. [self-, child-, and relational-focused mentalizing].

Another mother reflected about the situation at the referral for treatment:

*Every minute I offered another toy and then this and then that. And if she just sat still again for a minute I thought ‘oh she doesn’t like it. I have to offer something else again’. [I was] just so uncomfortable and uncertain. While sometimes it is fine if she is not playing for a while or if she wants to cuddle.* [self-, and child-focused mentalizing].

The capability to *regulate their own emotions, feelings and thoughts*, and in addition *to observe their child* closely, were mentioned as conditions for understanding the child.

As one mother mentioned when talking about her improvements with self-regulation:

*But just because I am becoming more comfortable myself and calmer, I think I am better able to observe her needs.*

Another mother said:

*Yes, in the beginning I found that [understanding the child] very difficult, but by sitting there in that [play]room with [the therapist], it got better and better and I thought ‘oh now she [the child] wants for example that toy or now she wants for example that.’ So by really watching her carefully it did become clearer to me.* [self-, and child-focused mentalizing].

Another mother learned by observation that the inner world of her son is different from her own:

*Learning to observe the way he thinks and not how I think he thinks*. [self, and child-focused mentalizing].

Some parents mentioned the power of *parental confidence*. The feeling of the bond with the child, and the confirmation in the child’s behavior of this bond, boosted their self-confidence as a parent.

As one mother explained:

*That he also responds well to me and I also respond clearly to him, which then gives a bond … and some more self-confidence as well. Yeah … like okay, he really is my child.* [self, and relation-focused mentalizing].

Another mother realized the reciprocity of mental states and behavior in the relationship with her daughter:

*Everything is a sort of connected. Self-confidence as a mother, that you can enjoy your daughter but also that she sees you as a mother, that you get a sort of a confirmation that you are doing well.* [self-, child- and relation-focused mentalizing].

In the following section the results will be discussed in the context of the research questions.

## Discussion

In this mixed method study we evaluate an integrated family approach in mental health care for patients and their children up to six years. The focus of our study was the quality of the parent-child interaction and parental mentalization and the aim was to investigate possible improvements on both of these after finishing treatment. The current study is part of a larger study in which we determined the key elements of an integrated family approach according to professionals who had conducted, and patients who had undertaken, this treatment. Furthermore, we examined the casefiles of patients and their children to determine the presence or absence of several problems (46).

Regarding our first research question, the expected improvement in the EA of the parent and the child in the parent-child interaction after a period of receiving an integrated family approach in treatment was found. Both the parent and child scales on the EAS showed significant and clinically relevant improvement: two-thirds of the participants showed a sufficiently positive interaction between parent and child at the post-measurement stage. The majority of the interviewed parents reported positive changes in the relationship with their children. They observed that the behavior of the child involved them (the parents) more, for example, by comfort-seeking behavior towards the parent. Regarding their own parental behavior they reported more sensitivity and non-intrusiveness. Most of them were able to illustrate these changes with consistent stories.

Regarding our second research question about changes in the parental perspective on the relationship with the child and the problems they experienced in child upbringing, we found that after treatment with an integrated family approach parents experienced significantly fewer problems in the relationship with their child as well as with the child’s upbringing. For more than half of the participants, there also was a clinically relevant change and for a part of them, scores at the post-measurement moved to the area for no to mild problems.

Regarding our third research question, if there was any improvement on parental reflective functioning for patients who had received treatment with an integrated family approach, we found mixed results. On the questionnaire measuring parental reflective functioning, we found a significant change in the certainty of mental states and only small changes in pre-mentalizing and interest and curiosity. At the individual level, the scores of a small number of participants showed clinical relevant change and movement toward the optimal score for certainty of mental states, and interest and curiosity. However, most of the interviewed parents reported improvements in mentalization by the awareness of the intentionality of their own behavior (self-focused mentalization) and the behavior of the child (child-focused mentalization), both dimensions of PRF (25–27). Furthermore, they showed in their narratives an understanding of how the relationship with their child was affected or shaped by processes and dynamics of intentional behavior in their interactions (27). In addition, parents showed an understanding of the interconnectedness between their improved self-regulation, observational skills, understanding of the child and their own behavior, and their sensitivity to the child’s needs. The latter, the interconnectedness of the capacity of mentalization and sensitivity to the child, is in line with previous research in which was statistically confirmed that these two were closely related (21).

Given the theoretical relationship between PRF and parental sensitivity, one might expect that improvements in sensitivity after treatment would also reflect an improvement in PRF. However, in our study this is only evident on the *certainty of mental states* scale of the PRFQ, and in what parents showed in the interviews. There may be several reasons for this. The PRFQ may not be sensitive enough to measure changes in a clinical population, or the study group was too small to reveal the changes. Another explanation may be in the differences of the two instruments which are not comparable, these being: observation scales of the parent-child interaction assessed by professionals, and a questionnaire which measures the parents’ opinion regarding statements about their functioning as a parent and giving meaning to their child’s behavior. Therefore, in future research of a clinical sample with a pre and post-measurement measuring PRF, another instrument would be more suitable, for instance the Parental Developmental Interview (22).

### Strengths and limitations

A strength of this study is that it was conducted in a naturalistic mental health setting reflecting everyday practice with a clinical sample. Because problems tend to cluster, these families often had to face multiple interrelated problems in different domains (parent, child, family, environment). These cases are often excluded from research due to heterogeneity in the variety of mental disorders, comorbidity, and a complexity of problems in the above mentioned domains. Most of the studies that investigate families with a complexity of problems focus only on specific aspects, while to come to a better understanding of this complexity, multiple perspectives (parents and children), and multiple constructs (individual, family, relationships) in research are necessary (47, 48).

Another strength of this study is the mixed methods design, in which observational data, questionnaires, and interviews were incorporated. This provides a broad range of information that is not often combined in research. Besides quantitative data, we used qualitative data from interviews of parents who have undertaken this treatment. A substantial percentage of parents of the whole sample of this study joined in these interviews. We believe that the voice of these parents provides deeper insight into the changes they have observed as a result of the treatment with an integrated family approach. In addition, it will serve as an illustration of what the quantitative outcomes of this study may refer to.

A limitation of this study is that we could not make any claims about causalities of the integrated family approach in treatment and the outcome, or which part of the treatment has been most beneficial to the results. This is because the treatment did not consist of a standard intervention, there was no control group, and it was a relatively small and heterogeneous sample, making comparison impossible. In addition, it cannot be ruled out that effects are due to spontaneous development, because no experimental design was used. However, in studying complex systems, as found in the real world, we need to adjust our research processes and set aside the expectation and the promise that we can provide clear and unambiguous answers about the relationships between phenomena or variables in a changing and uncontrollable context (47).

### Implications for clinical practice

What we have demonstrated in this study is that an integrated family approach in treatment in adult and child mental health care services contributes to more healthy interactions between parents and their young children. In addition, it provides parents with an increased confidence in parenthood, and satisfaction about the quality of the relationship with their child. Therefore, an integrated family approach in treatment provides a meaningful contribution in helping parents to break the cycle of intergenerational transmission of mental disorders and far-reaching adverse outcomes for the parent-child relationship and the development of their children.

## Conclusions

Treatment with an integrated family approach, in which the focus is on the mental disorder of the patient and simultaneously on parenthood, the young child and the developing parent-child relationship, can be a valuable addition to current practice. The enhancement of healthy parent-child interactions is essential for both the parent and the child, given the protective effects of a healthy parent-child relationship on the child’s development, and the experience of competence and satisfaction about parenting for the parent. Because of the reciprocal nature of the parent child-relationship it can contribute to positive cascading processes over time to parents and their young children.

## Data availability statement

The raw data supporting the conclusions of this article will be made available by the authors, without undue reservation.

## Ethics statement

The studies involving humans were approved by Medical Ethics Review Board at the University Medical Centre of Utrecht in the Netherlands (18-186/C). The studies were conducted in accordance with the local legislation and institutional requirements. Written informed consent for participation in this study was provided by the participants’ legal guardians/next of kin.

## Author contributions

HS: Conceptualization, Data curation, Formal analysis, Investigation, Methodology, Project administration, Resources, Supervision, Validation, Visualization, Writing – original draft, Writing – review & editing. LI: Conceptualization, Data curation, Formal analysis, Investigation, Methodology, Resources, Supervision, Validation, Visualization, Writing – original draft, Writing – review & editing. KD: Conceptualization, Methodology, Writing – review & editing. MS: Conceptualization, Methodology, Writing – review & editing.

## References

[1] Weissman MM Wickramaratne P Gameroff MJ Warner V Pilowsky D Kohad RG. Offspring of depressed parents: 30 years later. Am J Psychiatry. (2016) 173:1024–32. doi: 10.1176/appi.ajp.2016.15101327 27113122

[2] Uher R Pavlova B Radua J Provenzani U Najafi S Fortea L. Transdiagnostic risk of mental disorders in offspring of affected parents: A meta-analysis of family high-risk and registry studies. World Psychiatry. (2023) 22:433–48. doi: 10.1002/wps.21147 PMC10503921 37713573

[3] Stolper H van Doesum K Steketee M. How to support parents of infants and young children in mental health care: a narrative review. Front Psychol. (2021) 12:745800. doi: 10.3389/fpsyg.2021.745800 34867627 PMC8634941

[4] Stolper H van Doesum K Steketee M. Integrated family approach in mental health care by professionals from adult and child mental health services: A qualitative study. Front Psychiatry. (2022) 13:7815565. doi: 10.3390/ijerph192013164 PMC9096092 35573344

[5] Stolper H van Doesum K Henselmans P Bijl AL Steketee M. The patient’s voice as a parent in mental health care: A qualitative study. Int J Environ Res Public Health. (2022) 19:13164. doi: 10.3390/ijerph192013164 36293747 PMC9603497

[6] Stolper H van Doesum K Steketee M. An integrated family approach in the practice of adult and child mental health care. Front Psychiatry. (2024) 15:6. doi: 10.3389/fpsyt.2024.1298268 PMC11056573 38686126

[7] Field T. Maternal depression effects on infants and early interventions. Prev Med. (1998) 27:200–3. doi: 10.1006/pmed.1998.0293 9578995

[8] Forman DR O'hara MW Stuart S Gorman LL Larsen KE Coy KC. Effective treatment for postpartum depression is not sufficient to improve the developing mother–child relationship. Dev Psychopathol. (2007) 19:585–602. doi: 10.1017/S0954579407070289 17459185

[9] Thanhäuser M Lemmer G de Girolamo G Christiansen H. Do preventive interventions for children of mentally ill parents work? Results of a systematic review and meta-analysis. Curr Opin Psychiatry. (2017) 30:283–99. doi: 10.1097/YCO.0000000000000342 28505032

[10] Seifer R. “Young children with mentally ill parents: Resilient developmental systems.” In: Resilience and vulnerability: Adaptation in the context of childhood adversities. Cambridge University Press. (2003). p. 29–49. doi: 10.1017/CBO9780511615788.004

[11] Harder S Davidsen K MacBeth A Lange T Minnis H Andersen MS. Wellbeing and resilience: mechanisms of transmission of health and risk in parents with complex mental health problems and their offspring—The WARM Study. BMC Psychiatry. (2015) 15:1–13. doi: 10.1186/s12888-015-0692-6 26654720 PMC4674908

[12] Aktar E Qu J Lawrence PJ Tollenaar MS Elzinga BM Bögels SM. Fetal and infant outcomes in the offspring of parents with perinatal mental disorders: earliest influences. Front Psychiatry. (2019) 10:391. doi: 10.3389/fpsyt.2019.00391 31316398 PMC6610252

[13] Barnes J Theule J. Maternal depression and infant attachment security: A meta-analysis. Infant Ment Health J. (2019) 40:817–34. doi: 10.1002/imhj.21812 31415711

[14] Schechter DS Willheim E. Disturbances of attachment and parental psychopathology in early childhood. Child Adolesc Psychiatr Clinics. (2009) 18:665–86. doi: 10.1016/j.chc.2009.03.00 PMC2690512 19486844

[15] McGoron L Gleason MM Smyke AT Drury SS Nelson CA III Gregas MC. Recovering from early deprivation: attachment mediates effects of caregiving on psychopathology. J Am Acad Child Adolesc Psychiatry. (2012) 51:683–93. doi: 10.1016/j.jaac.2012.05.004 PMC4127424 22721591

[16] Doty JL Davis L Arditti JA. Cascading resilience: Leverage points in promoting parent and child well-being. J Family Theory Rev. (2017) 9:111–26. doi: 10.1111/jftr.12175

[17] Wan MW Green J. The impact of maternal psychopathology on child-mother attachment. Arch Womens Ment Health. (2009) 12:123–34. doi: 10.1007/s00737-009-0066-5 19337701

[18] Biringen Z Derscheid D Vliegen N Closson L Easterbrooks MA. Emotional availability (EA): Theoretical background, empirical research using the EA Scales, and clinical applications. Dev Rev. (2014) 34:114–67. doi: 10.1016/j.dr.2014.01.002

[19] Sameroff AJ. Ports of Entry and the Dynamics of Mother-Infant Interventions. Guilford publications, New York. (2004).

[20] Fonagy P Allison E. The role of mentalizing and epistemic trust in the therapeutic relationship. Psychotherapy. (2014) 51:372. doi: 10.1037/a0036505 24773092

[21] Zeegers MAJ Colonnesi C Stams G-JJM Meins E. Mind matters: A meta-analysis on parental mentalization and sensitivity as predictors of infant–parent attachment. psychol Bull. (2017) 143:1245. doi: 10.1037/bul0000114 28805399

[22] Slade A. Parental reflective functioning: An introduction. Attachment Hum Dev. (2005) 7:269–81. doi: 10.1080/14616730500245906 16210239

[23] Sharp C Fonagy P. The parent's capacity to treat the child as a psychological agent: Constructs, measures and implications for developmental psychopathology. Soc Dev. (2008) 17:737–54. doi: 10.1111/j.1467-9507.2007.00457.x

[24] Luyten P Mayes LC Nijssens L Fonagy P. The parental reflective functioning questionnaire: Development and preliminary validation. PloS One. (2017) 12:e0176218. doi: 10.1371/journal.pone.0176218 28472162 PMC5417431

[25] Suchman NE DeCoste C Leigh D Borelli J. Reflective functioning in mothers with drug use disorders: Implications for dyadic interactions with infants and toddlers. Attachment Hum Dev. (2010) 12:567–85. doi: 10.1080/14616734.2010.501988 PMC2953729 20931415

[26] Borelli JL St John HK Cho E Suchman NE. Reflective functioning in parents of school-aged children. Am J Orthopsychiatry. (2016) 86:24. doi: 10.1037/ort0000141 26618938 PMC5102156

[27] Smaling HJA Huijbregts SCJ van der Heijden KB van Goozen SHM Swaab H. Maternal reflective functioning as a multidimensional construct: Differential associations with children’s temperament and externalizing behavior. Infant Behav Dev. (2016) 44:263–74. doi: 10.1016/j.infbeh.2016.06.007 27522031

[28] Fonagy P Gergely G Jurist EL eds. Affect regulation, mentalization and the development of the self. Routledge, Oxfordshire (2018).

[29] Bakermans-Kranenburg MJ Van Ijzendoorn MH Juffer F. Less is more: meta-analyses of sensitivity and attachment interventions in early childhood. psychol Bull. (2003) 129:195. doi: 10.1037/0033-2909.129.2.195 12696839

[30] Nijssens L Vliegen N Luyten P. The mediating role of parental reflective functioning in child social–emotional development. J Child Family Stud. (2020) 29:2342–54. doi: 10.1007/s10826-020-01767-5

[31] Nijssens L Luyten P Bales DL. Mentalization-based treatment for parents (MBT-P) with borderline personality disorder and their infants. In: Midgley N. Vrouva I. (Eds.), Minding the child: Mentalization-based interventions with children, young people and their families. Routledge/Taylor & Francis Group. (2012), 79–97.

[32] Biringen Z. The Emotional Availability (EA) Scales 4th edition and the Emotional Attachment & Emotional Availability (EA2) Clinical Screener: Infancy/early childhood version; middle childhood/youth versions; therapist/interventionist manual; couple relationship manual. Boulder, CO: international Center for Excellence in Emotional Availability (2008).

[33] Licata M Paulus M Thoermer C Kristen S Woodward AL Sodian B. Mother–infant interaction quality and infants' ability to encode actions as goal-directed. Soc Dev. (2014) 23:340–56. doi: 10.1111/sode.12057

[34] Nicolson S Carron S-P Campbell P. Supporting early infant relationships and reducing maternal distress with the Newborn Behavioral Observations: A randomized controlled effectiveness trial. Infant Ment Health J. (2022) 43:455–73. doi: 10.1002/imhj.21987 PMC9324818 35531961

[35] Endendijk JJ Groeneveld MG Deković M Van Den Boomen C. Short-term test–retest reliability and continuity of emotional availability in parent–child dyads. Int J Behav Dev. (2019) 43:271–7. doi: 10.1177/0165025419830256

[36] Vermulst Ad Kroes G De Meyer R Nguyen L Veerman JW. User Manual PSQ. Praktikon, Nijmegen (2015).

[37] Egberink IJL De Leng WE Vermeulen CSM. COTAN beoordeling 2017, OpvoedingsBelasting Vragenlijst. Available at: https://www.cotandocumentatie.nl (Accessed January 25, 2024).

[38] Camoirano A. Mentalizing makes parenting work: A review about parental reflective functioning and clinical interventions to improve it. Front Psychol. (2017) 8. doi: 10.3389/fpsyg.2017.00014 PMC5247433 28163690

[39] Anis L Perez G Benzies KM Ewashen C Hart M Letourneau N. Convergent validity of three measures of reflective function: Parent development interview, parental reflective function questionnaire, and reflective function questionnaire. Front Psychol. (2020) 11:574719. doi: 10.3389/fpsyg.2020.574719 33391088 PMC7772143

[40] Cooke D Priddis L Luyten P Kendall G Cavanagh R. Paternal and maternal reflective functioning in the Western Australian Peel child health study. Infant Ment Health J. (2017) 38:561–74. doi: 10.1002/imhj.21664 28833359

[41] Pazzagli C Delvecchio E Raspa V Mazzeschi C Luyten P. The parental reflective functioning questionnaire in mothers and fathers of school-aged children. J Child Family Stud. (2018) 27:80–90. doi: 10.1007/s10826-017-0856-8

[42] Madsen EB Væver MS Egmose I Krogh MT Haase TW de Moor MHM. Parental reflective functioning in first-time parents and associations with infant socioemotional development. J Child Family Stud. (2023), 1–13. doi: 10.1007/s10826-023-02565-5

[43] Faul F Erdfelder E Lang A-G Buchner A. G* Power 3: A flexible statistical power analysis program for the social, behavioral, and biomedical sciences. Behavior Research Methods. (2007) 39:175–91. doi: 10.3758/BF03193146 17695343

[44] Jacobson NS Truax P. Clinical significance: a statistical approach to defining meaningful change in psychotherapy research. Journal of Consulting and Clinical Psychology (1991) 59:12–19. doi: 10.1037//0022-006x.59.1.12 2002127

[45] Maassen GH. The standard error in the Jacobson and Truax Reliable Change Index: The classical approach to the assessment of reliable change. J Int Neuropsychol Soc. (2004) 10:888–93. doi: 10.1017/S1355617704106097 15637779

[46] Stolper H van der Vegt M van Doesum K Steketee M. The Integrated Family Approach in Mental Health Care Services: A Study of Risk Factors. International Journal of Environmental Research and Public Health (2024) 21(5):640. doi: 10.3390/ijerph21050640 38791854 PMC11121543

[47] Boulton JG Allen PM Bowman C. Embracing complexity: Strategic perspectives for an age of turbulence. Oxford University Press, Oxford (2015). doi: 10.1093/acprof:oso/9780199565252.001.0001

[48] Bodden DHM Deković M. Multiproblem families referred to youth mental health: What's in a name? Family process. (2016) 55:31–47. doi: 10.1111/famp.12144 25754003

